# Molecular epidemiology of giardiasis among Orang Asli in Malaysia: application of the triosephosphate isomerase gene

**DOI:** 10.1186/1471-2334-14-78

**Published:** 2014-02-12

**Authors:** Tengku Shahrul Anuar, Siti Nor Azreen, Fatmah Md Salleh, Norhayati Moktar

**Affiliations:** 1 Department of Medical Laboratory Technology, Faculty of Health Sciences, Universiti Teknologi MARA, Puncak Alam Campus, 42300 Selangor, Malaysia; 2 Department of Parasitology and Medical Entomology, Faculty of Medicine, Universiti Kebangsaan Malaysia, Jalan Raja Muda Abdul Aziz, 50300 Kuala Lumpur, Malaysia

**Keywords:** *Giardia duodenalis*, Assemblage, Risk factors, Orang Asli, Malaysia

## Abstract

**Background:**

*Giardia duodenalis* is a flagellate parasite which has been considered the most common protozoa infecting human worldwide. Molecular characterization of *G. duodenalis* isolates have revealed the existence of eight groups (Assemblage A to H) which differ in their host distribution. Assemblages A and B are found in humans and in many other mammals.

**Methods:**

This cross-sectional study was conducted to identify assemblage’s related risk factors of *G. duodenalis* among Orang Asli in Malaysia. Stool samples were collected from 611 individuals aged between 2 and 74 years old of whom 266 were males and 345 were females. Socioeconomic data were collected through a pre-tested questionnaire. All stool samples were processed with formalin-ether sedimentation and Wheatley’s trichrome staining techniques for the primary identification of *G. duodenalis*. Molecular identification was carried out by the amplification of a triosephosphate isomerase gene using nested-PCR assay.

**Results:**

Sixty-two samples (10.2%) were identified as assemblage A and 36 (5.9%) were assemblage B. Risk analysis based on the detected assemblages using univariate and logistic regression analyses identified subjects who have close contact with household pets *i.e.* dogs and cats (OR = 2.60; 95% CI = 1.42, 4.78; *P* = 0.002) was found to be significant predictor for assemblage A. On the other hand, there were three significant risk factors caused by assemblage B: (i) children ≤15 years old (OR = 2.33; 95% CI = 1.11, 4.87; *P* = 0.025), (ii) consuming raw vegetables (OR = 2.82; 95% CI = 1.27, 6.26; *P* = 0.011) and (iii) the presence of other family members infected with giardiasis (OR = 6.31; 95% CI = 2.99, 13.31; *P* < 0.001).

**Conclusions:**

The present study highlighted that *G. duodenalis* infection among Orang Asli was caused by both assemblages with significant high prevalence of assemblage A. Therefore, taking precaution after having contact with household pets and their stool, screening and treating infected individuals, awareness on the importance of good health practices and washing vegetables are the practical intervention ways in preventing giardiasis in Orang Asli community.

## Background

*Giardia* is a genus of intestinal flagellates that infects a wide range of vertebrate hosts. The genus currently comprises six species, namely *Giardia agilis* in amphibians, *Giardia ardeae* and *Giardia psittaci* in birds, *Giardia microti* and *Giardia muris* in rodents and *Giardia duodenalis* in mammals. These species are distinguished on the basis of the morphology and ultrastructure of their trophozoite [1]. *Giardia duodenalis* (syn. *Giardia intestinalis* and *Giardia lamblia*) is the only species within the genus *Giardia* that infects humans, although it is also found in other mammals including pets and livestock [2]. The infection has a global distribution, with an estimated 2.8 × 10^8^ cases per year, represents the most common gastrointestinal parasitic infection of humans in developed countries [3]. In Asia, Africa and Latin America, about 200 million people have symptomatic giardiasis with some 500,000 new cases reported each year [4]. There are four characteristics of *G. duodenalis* that influence the epidemiology of infection: (i) the infective dose is about 10 to 100 cysts in humans; (ii) cysts are immediately infectious when excreted in stool and can be transmitted by human-to-human or animal-to-animal contact; (iii) cysts are remarkably stable and can survive for weeks to months in the environment and (iv) environmental contamination can lead to the contamination of drinking water and food [5,6].

A considerable amount of data has shown that *G. duodenalis* should be considered a species complex whose members show little variation in their morphology yet can be assigned to at least eight distinct assemblages (A to H) based on genetic analyses [7,8]. The analysis of more than a thousand human isolates from different geographical locations, examined by PCR amplification of DNA extracted directly from stool has demonstrated that in almost all cases, only *G. duodenalis* assemblages A and B are associated with human infections [5]. The prevalence of each assemblage varies considerably from country to country; assemblage B seems more common overall, but no strong conclusions can be drawn from current data. The remaining assemblages (C to G) are likely to be host specific, as assemblages C and D have been identified in dogs, cats, coyotes and wolves; assemblage E in cattle, sheep, goats, pigs, water buffaloes and muflons; assemblage F in cats and assemblage G in rats.

In Malaysia, giardiasis is an endemic disease and is associated with malnutrition among children in the rural areas resulting in stunting, wasting and vitamin A deficiency [9,10]. The prevalence of giardiasis varies between 0.2 to 20% [11-13]. Most of the epidemiological studies detected *G. duodenalis* on the basis of microscopic examination without employing molecular approach. Data on genotypes of *G. duodenalis* up to the assemblage level remains scarce. In a previous genotyping study using SSU rRNA locus, one specimen was identified as assemblage A in 42 specimens and the rest were assemblage B [14]. In a study on immunocompromised patients, assemblage A was identified in four of the microscopy-positive specimens using triosephosphate isomerase (*tpi*) gene [15]. Assemblage A was also isolated from environmental samples including recreational lake water and water bodies in a zoo [16,17]. In addition, genotyping study was conducted on animals and assemblages A and E were detected among goats [18]. Recent study conducted by Huey *et al.*[19] based on multilocus analysis revealed that 42% of the Orang Asli isolates belong to assemblage A and 45% belonged to assemblage B. However, determining the association of potential risk factors caused by both assemblages were not conducted in the previous studies which limit our understanding on the dynamic transmissions and the source of *G. duodenalis* infection in this country. Thus, the present study was conducted to identify *G. duodenalis* assemblage and the risk factors based on *tpi* gene to attain better understanding of the genetic diversity and transmission of giardiasis. The *tpi* gene was chosen because of the high genetic heterogeneity displayed by *Giardia* species at this locus [20-22].

## Methods

### Study area and design

The cross-sectional study was conducted from June to December 2011 among 611 individuals living in eight villages from Negeri Sembilan, Perak and Pahang of Malaysia. Sample selection was achieved using a two-stage sampling method: (i) random selection of villages (ii) random selection of 10 to 15 households per village. All village entry has been approved by the Ministry of Rural and Regional Development of Malaysia. With an expected minimum prevalence of *G. duodenalis* in the study area was 20% [13], the 95% confidence interval and an absolute precision of 0.05 [23], the appropriate sample size for the study was estimated to be 246 subjects. Within each village, subjects over 2 years of age and those who provided consent to participate were included in this study. Exclusion criteria included children below 2 years old and refusal to participate.

### Structured questionnaire

The rationale and procedures of the study were explained and an informed consent sheet was signed by the head of the household or a designated literate substitute. A trained research team interviewed each subject using a previously tested, structured questionnaire that sought information on the following groups of variables: (i) demographic data (*i.e.* age, gender and education level); (ii) socioeconomic background (*i.e.* occupation, household income and educational status); (iii) behavioural risks (*i.e.* personal hygiene such as hand washing and food consumption); (iv) environmental sanitation and characteristics of living condition (*i.e.* types of water supply, latrine system and sewage disposal system); (v) close contact with household pets (*i.e.* dogs or cats). This questionnaire was first designed in English and then translated and pretested in the Malay language, which is the national language for Malaysia and well understood by the subjects. For children, the questionnaire was completed by interviewing their parents or guardians who signed the informed consent. Subjects who participated in this study were honoured with a small token of appreciation.

### Stool samples collection

Following the administration of the questionnaire, a 100 ml wide mouth screw-capped container pre-labelled with the subject’s name and code were distributed to all subjects for the collection of their stool sample the next day. Their ability to recognize their names was counter-checked. Each subject was instructed to scoop a thumb size stool sample using a provided scoop into the container. Then, the container was placed in a zip-locked plastic bag. Parents and guardians were instructed to monitor their children during the sample collection to ensure that they place their stool samples into the right containers. All study subjects were asked to provide sufficiently large stool sample (at least 10 g) so that both microscopic techniques and the molecular method could be performed. This study had to rely on a single stool collection because of the limitation of resources and the cultural belief of the Orang Asli against giving away their stool samples.

### Parasitological examination

Stool samples were processed in the designated area of work in the study village within a minimum of four hours after collection by experienced laboratory technicians. Approximately, 5 g of each stool sample were kept in a 15 ml centrifuge tube containing 3 ml Polyvinyl Alcohol (PVA). PVA-fixed samples were forwarded to the Parasitology Department of the Faculty of Medicine, Universiti Kebangsaan Malaysia. The samples were processed using the Wheatley’s trichrome staining method. Briefly, the smear cover slips were stained as follows: (i) iodine alcohol (15 min); (ii) 70% alcohol (10 min); (iii) Wheatley’s trichrome stain (10 minutes); (iv) acid alcohol (3 s); (v) 95% alcohol (5 min); (vi) absolute alcohol (5 min); (vii) Wintergreen oil (5 min) [24]. Each cover slip was mounted using Distrene, Plasticiser and Xylene (DPX) and examined under the light microscope at a magnification of 1,000×. Additionally, another half of the samples were kept unpreserved and stored at 4°C upon arrival at the laboratory for further analysis by formalin-ether sedimentation and DNA extraction. Briefly, 2 g of stool sample were mixed with 7 ml of formalin and 3 ml of ether, centrifuged, stained with Lugol’s iodine, and finally examined under light microscopy at a magnification of 400× [25]. Samples were considered microscopically positive if cysts and/or trophozoites were detected in at least one of the two techniques, and negative if negative in both the techniques. All microscopically-positive (n = 110) samples were further characterized using molecular procedures.

### DNA extraction

DNA was extracted directly from all stool samples using QIAamp Stool DNA extraction kit (Qiagen, Hilden, Germany) according to the manufacturer’s instructions. Briefly, 0.2 g of stool was placed in a microcentrifuge tube, incubated at 70°C for 5 min with the cell lysis and disruption agent provided by the manufacturer. This was then subjected to homogenization and lysis procedure for complete cell lysis by using mechanical vortex (Silent Crusher S, Germany). The final DNA elution was prepared in 70 μl of elution buffer. The concentration of extracted DNA was measured by a spectrophotometer (Eppendorf, Germany) at 260 nm and then the samples were stored at-20°C until used.

### PCR amplification of the triosephosphate isomerase gene

A partial sequence of *tpi* gene (530-bp) was amplified using nested-PCR protocol according to Sulaiman *et al.*[22]. Primary PCR was run using forward primer AL3543 (5′-AAA TIA TGC CTG CTC GTC G-3′) and reverse primer AL3546 (5′-CAA ACC TTI TCC GCA AAC C-3′). For secondary PCR, forward primer AL3544 (5′-CCC TTC ATC GGI GGT AAC TT-3′) and reverse primer AL3545 (5′-GTG GCC ACC ACI CCC GTG CC-3′) were used. Primary and secondary PCRs were performed in a 50 μl PCR mix comprising 0.2 μM of each primer (Bio Basic Canada Inc). 1 U of HotStarTaq® *Plus* DNA Polymerase (Qiagen, Hilden, Germany), 1 × PCR buffer (Qiagen, Hilden, Germany), 200 μM dNTP (Fermentas, Ontario, Canada), 1.5 mM MgCl_2_ (Qiagen, Hilden, Germany) and 0.2 mg/ml BSA (New England Biolabs, Ipswich, USA). 2 μl of DNA template were used and the prepared master mix was incubated in the Eppendorf Pro-S thermal cycler (Hamburg, Germany) under the following conditions: initial hot start at 95°C for 5 min, 35 amplification cycles at 94°C for 45 s, 50°C for 45 s (58°C for secondary PCR), 72°C for 60 s and a final extension at 72°C for 10 min.

In addition, the first PCR product of the reaction described by Sulaiman *et al.*[22] underwent further amplification using a set of separate A [26] and B [27] assemblage-specific primers. Presence of mixed infection was detected by visualizing the occurrence of bands in the agarose gel at 332-bp for assemblage A amplified using primers AssAF (5′-CGC CGT ACA CCT GTC-3′) and AssAR (5′-AGC AAT GAC AAC CTC CTT CC-3′) and at 400-bp for assemblage B amplified using primers AssBF (5′-GTT GTT GTT GCT CCC TCC TTT-3′) and AssBR (5′-CCG GCT CAT AGG CAA TTA CA-3′). The PCR reaction mix consisted of 0.2 μM (0.4 μM for assemblage B) of each primer (Bio Basic Canada Inc). 1.25 U of HotStarTaq® Plus DNA Polymerase (Qiagen, Hilden, Germany), 1 × PCR buffer (Qiagen, Hilden, Germany), 200 μM dNTP (Fermentas, Ontario, Canada), 1.5 mM MgCl_2_ (Qiagen, Hilden, Germany) and 0.1 mg/ml BSA (New England Biolabs, Ipswich, USA) to a final volume of 25 μl. 1 μl of DNA template was added for assemblage A and 2 μl was added for assemblage B for the PCR amplifications following the cycle parameter: initial hot start at 95°C for 5 min, initial denaturation at 94°C for 10 min and 35 amplification cycles at 94°C for 45 s, 64°C for 45 s (62°C for secondary PCR) and 72°C for 45 s.

In all the PCR reactions, a *Giardia*-positive DNA sample and distilled water were used as a positive and negative control. The amplified products were analyzed by electrophoresis in 1.5% agarose gel (Vivantis) and stained with GelRed (0.1 μl/mL: Biotium).

### DNA sequencing and phylogenetic analysis

The positive amplicons were then purified using the SolGent™ kit (South Korea) according to the manufacturer’s instruction. All purified amplicons were sequenced in both directions using the same primer sets as in the respective PCR assay with an ABI 3730XL sequencer (Applied Biosystems, USA). The chromatograms and sequences generated from this study were viewed and assembled using the BioEdit Sequence Alignment Editor Programme (http://www.mbio.ncsu.edu/bioedit/bioedit.html). Preliminary similarity comparison of the consensus sequence with the sequences in GenBank database was made using Basic Local Alignment Search Tool (BLAST) (http://www.ncbi.nlm.nih.gov/BLAST). The isolate sequences were genotyped into assemblage using multiple alignments implemented by ClustalW [28] with previously defined reference sequences retrieved from GenBank database. The following reference sequences were used in the analysis: AF069556 (assemblage A), AF069557 (assemblage A), L02120 (assemblage A), U57897 (assemblage A), AF069560 (assemblage B), AF069561 (assemblage B), L02116 (assemblage B), AF069563 (assemblage C) and AF069559 (assemblage E).

Phylogenetic analysis was performed in MEGA 5 (http://www.megasoftware.net) using neighbour-joining algorithms with evolutionary distances calculated by Kimura-2-parameter method [29] and 1000 bootstrap value. Sequences of *G. ardeae* (AF069564) and *G. muris* (AF069565) were used as the out group since the construction on an unrooted tree showed them to be the most divergent members under analysis.

### Data analysis

Data was entered in a Microsoft Access and was cross-checked by technical staff to ensure that they were entered correctly. Statistical analysis was performed using the SPSS software (Statistical Package for the Social Sciences) program for Windows version 20 (SPSS, Chicago, IL, USA). Prevalence of *G. duodenalis* assemblages A and B were determined on the basis of microscopic examination and the molecular method. Only those subjects with complete questionnaire data and whose stool samples were processed via formalin-ether sedimentation, Wheatley’s trichrome staining and nested-PCR assay were included in the final analyses.

For descriptive analysis, percentage rates were used to describe the characteristics of the studied population, including the prevalence of *G. duodenalis* assemblage A and assemblage B. A Chi-square test (χ^2^) was used to test the association between the variables. In the univariate analysis, the dependent variable was prevalence of *G. duodenalis* assemblage A and assemblage B, while the independent variables were demographic and socioeconomic factors, behavioural risks, environmental sanitation, living condition characteristics and close contact with household pets. All univariate models were used to assess potential associations between *G. duodenalis* assemblages A and B infections and the characteristics of the potential associated factor. The level of statistical significance was set at *P* < 0.05 and for each statistically significant factor, an odds ratio (OR) and 95% confidence interval (CI) were computed for both univariate and multivariate logistic regression analysis. All factors that were significant in the univariate model were included in a logistic multivariate analysis to determine which factors could be dropped from the multivariable model.

### Ethical considerations and treatment

The study protocol (Reference Number: UKM 1.5.3.5/244/FF-165-2011) was reviewed and approved by the Ethics Committee of Universiti Kebangsaan Malaysia Medical Centre (UKMMC) and permission for field work was obtained from the Ministry of Rural and Regional Development of Malaysia before starting the study. Village meetings were held and village authorities and villagers were handed detailed explanations about the aims, procedures, potential risks and benefit from the study. During the meetings, they were also informed that their identities and personal particulars would be kept strictly confidential and they could withdraw from the study at any point of time without citing reasons for doing so. If they agreed to participate, their consent was obtained in written form (signature or thumbprint for those who were illiterate) or parents were approached for consent on behalf of their children. At the end of the study, each subject confirmed with *G. duodenalis* infection by PCR was treated with metronidazole according to the Ministry of Health Malaysia, free of charge.

## Results

### Characteristics of the study population

Single stool samples were randomly collected from a total of 611 subjects. With regards to the age groups, 277 (45.3%) were less than 15 years while 334 (54.7%) were 15 years old or above (≥15), with a median age of 18 years [interquartile range (IQR) 9-34]. Subjects who participated in this study comprised 266 (43.5%) males and 345 (56.5%) females.

More than half (68%) of the parents have low level of education i.e., less than 6 years of formal education. The majority of the parents did odd jobs such as selling forest products without any stable income. Some were daily wage earners working in rubber or palm oil plantations, unskilled labourers in factories or construction sites. Therefore, 51.6% of the households belonged to people who earned less than RM500 per month (≤US$156.02), the poverty income threshold in Malaysia (Department of Statistics Malaysia, 1997. Profile of Orang Asli in Peninsular Malaysia, Kuala Lumpur) which is inadequate to maintain a good living standard. Although 61.9% if the houses have provision of basic infrastructure such as treated water supply and 71.4% have pour flush toilet, at least 38.1% are still using untreated water originating from a nearby river for their domestic needs and 28.6% still defecate indiscriminately in the river or bush. More than half of the households (55.8%) kept dogs and cats as their pets. Most of these pets are left to roam freely. The villagers have very close contact with the dogs and cats. Occasionally, these pets also slept, defecated indoors and accompanied the villagers into the forest to harvest forest products.

### Prevalence of *Giardia duodenalis* assemblages A and B infections

Table 1 shows that 10.2% (62/611) and 5.9% (36/611) of the subjects were infected with *G. duodenalis* assemblage A and assemblage B, respectively. The prevalence of *G. duodenalis* assemblages A and B infections were not significantly associated with gender. However, the prevalence of *G. duodenalis* assemblage B infection was significantly higher in the younger age group of less than 15 years (*P* = 0.021).

**Table 1:** **Distribution of *Giardia duodenalis *** **assemblage A and assemblage B by age and gender among Orang Asli in Malaysia (n = 611)**

**Variables**	**No. examined**	**Assemblage A**		**Assemblage B**	
		**No. infected**	**% Infected**	**No. infected**	**% Infected**
Age groups (years)					
<15	277	28	10.1	23	8.3
≥15	334	34	10.1	13	3.9
Gender					
Male	266	32	12.0	17	6.4
Female	345	30	8.7	19	5.5
Total	611	62	10.2	36	5.9

### Associated factors for *Giardia duodenalis* assemblages A and B infections

The association of *G. dudoenalis* assemblages A and B infections and sociodemographic characteristics are shown in Table 2. The results showed that drinking untreated water (OR = 1.72; 95% CI = 1.01, 2.91; *P* = 0.042) and close contact with household pets (OR = 2.72; 95% CI = 1.48, 4.98; *P* = 0.001) were significantly associated with *G. duodenalis* assemblage A infection. On the other hand, *G. duodenalis* assemblage B infection was associated with five factors which include children less than 15 years old (OR = 2.23; 95% CI = 1.11, 4.50; *P* = 0.021), consuming raw vegetables (OR = 3.13; 95% CI = 1.48, 6.61; *P* = 0.002), eating fresh fruits (OR = 3.47; 95% CI = 11.50, 1.51; *P* = 0.030), non working mother (OR = 3.07; 95% CI = 1.105, 9.04; *P* = 0.033) and the presence of other family members infected with giardiasis (OR = 7.75; 95% CI = 3.76, 15.94; *P* < 0.001).

**Table 2:** **Potential risk factors associated with *Giardia duodenalis *** **assemblages A and B infections among Orang Asli (univariate analysis, n = 611)**

	**Assemblage A**				**Assemblage B**			
**Variables**	**No. examined**	**% Infected**	**OR (95% CI)**	***P*-value**	**No. examined**	**% Infected**	**OR (95% CI)**	***P*-value**
Age (years)								
<15	277	10.1	0.99 (0.759, 1.68)	0.977	277	8.3	2.23 (1.11, 4.50)	0.021^a^
≥15	334	10.2	1		334	3.9	1	
Gender								
Male	266	12.0	1.44 (0.85, 2.43)	0.176	266	6.4	1.17 (0.60, 2.30)	0.646
Female	345	8.7	1		345	5.5	1	
Drinking untreated water								
Yes	233	13.3	1.72 (1.01, 2.91)	0.042^a^	233	8.2	1.89 (0.96, 3.71)	0.062
No	378	8.2	1		378	4.5	1	
Bathing and washing in the river								
Yes	175	7.4	0.63 (0.34, 1.20)	0.159	175	8.0	1.64 (0.82, 3.28)	0.161
No	436	11.2	1		436	5.0	1	
Not washing hands after playing with soil or gardening								
Yes	215	10.7	1.10 (0.64, 1.89)	0.740	215	10.7	1.10 (0.64, 1.89)	0.740
No	396	9.8	1		396	9.8	1	
Close contact with household pets								
Yes	341	13.8	2.72 (1.48, 4.98)	0.001^a^	341	7.0	1.63 (0.80, 3.32)	0.176
No	270	5.6	1		270	4.4	1	
	**Assemblage A**				**Assemblage B**			
**Variables**	**No. examined**	**% Infected**	**OR (95% CI)**	***P*-value**	**No. examined**	**% Infected**	**OR (95% CI)**	***P*-value**
Indiscriminate defecation								
Yes	205	10.7	1.10 (0.64, 1.91)	0.734	205	7.3	1.45 (0.73,2.87)	0.288
No	406	9.9	1		406	5.2	1	
Sewage disposal								
Outdoor	264	10.2	1.01 (0.60, 1.73)	0.954	264	7.6	1.70 (0.86, 3.34)	0.123
Common drainage	347	10.1	1		347	4.6	1	
Eating with hands								
Yes	390	9.2	0.76 (0.45, 1.30)	0.319	390	6.7	1.51 (0.71, 3.19)	0.280
No	221	11.8	1		221	4.5	1	
Consuming raw vegetables								
Yes	287	11.8	1.42 (0.84, 2.41)	0.190	287	9.1	3.13 (1.48, 6.61)	0.002^a^
No	324	8.6	1		324	3.1	1	
Eating fresh fruits								
Yes	470	10.4	1.15 (0.60, 2.18)	0.678	470	7.0	3.47 (1.05, 11.50)	0.030^a^
No	141	9.2	1		141	2.1	1	
Father’s education								
Non-educated (<6 yrs)	253	9.9	1.02 (0.48, 2.15)	0.965	253	4.3	0.98 (0.33, 2.90)	0.973
Educated (>6 yrs)	113	9.7	1		113	4.4	1	
Mother’s education								
Non-educated (<6 yrs)	250	10.8	1.44 (0.65, 3.17)	0.363	250	4.0	0.76 (0.27, 2.16)	0.610
Educated (>6 yrs)	116	7.8	1		116	5.2	1	
Non working mother								
Yes	157	10.8	1.21 (0.61, 2.42)	0.581	157	7.0	3.07 (1.05, 9.04)	0.033^a^
No	209	9.1	1		209	2.4	1	
	**Assemblage A**				**Assemblage B**			
**Variables**	**No. examined**	**% Infected**	**OR (95% CI)**	***P*-value**	**No. examined**	**% Infected**	**OR (95% CI)**	***P*-value**
Household members								
≥8	216	11.1	1.17 (0.68, 2.02)	0.560	216	6.5	1.18 (0.59, 2.35)	0.647
<8	395	9.6	1		395	5.6	1	
Household monthly income^b^								
≤RM500	315	10.2	1.00 (0.59, 1.70)	0.992	315	7.3	1.72 (0.85, 3.45)	0.127
>RM500	296	10.1	1		296	4.4	1	
Other family members infected with giardiasis								
Yes	142	14.1	1.67 (0.94, 2.95)	0.076	142	16.9	7.75 (3.76, 15.94)	<0.001^a^
No	469	9.0	1		469	2.6	1	

CI = Confidence interval.

OR = Odds ratio, Reference group marked as OR = 1.

^a^Significant association (*P* < 0.05).

^b^RM = Malaysian Ringgit; ≤US$ 156.02 (10^th^ December 2013).

### Aetiological factors associated with *Giardia duodenalis* assemblage A and assemblage B infections

Logistic regression analysis confirmed that individuals who have close contact with household pets i.e., dogs and cats were 2.6 times (95% CI = 1.42, 4.78; *P* = 0.002) more likely to be infected with *G. duodenalis* assemblage A as compared to those who do not keep dogs and cats as their pets. In addition, children less than 15 years old (OR = 2.33; 95% CI = 1.11, 4.87; *P* = 0.025), those being a consumer of raw vegetables (OR = 2.82; 95% CI = 1.27, 46.26; *P* = 0.011) and presence of other family members infected with giardiasis (OR = 6.31; 95% CI = 2.99, 13.31; *P* < 0.001) were more likely to be infected with *G. duodenalis* assemblage B (Table 3).

**Table 3:** **Odds ratio (OR) of the aetiological factors for *Giardia duodenalis *** **assemblages A and B infections among Orang Asli**

**Variables**	**OR**	**95% CI**	***P*-value**
Assemblage A			
Close contact with household pets	2.60	1.42, 4.78	0.002
Assemblage B			
Children (≤15 years)	2.33	1.11, 4.87	0.025
Consuming raw vegetables	2.82	1.27, 6.26	0.011
Presence of other family members infected with *G. duodenalis*	6.31	2.99, 13.31	<0.001

CI = Confidence interval.

### Molecular characterization of *Giardia duodenalis* isolate

**Figure 1:**
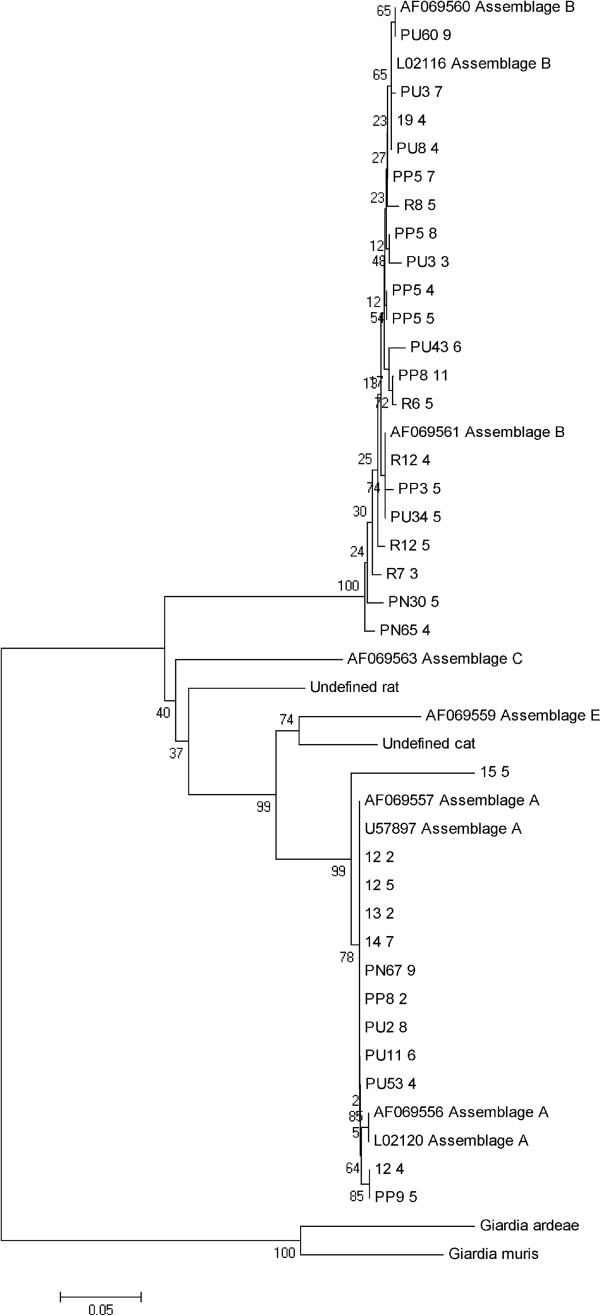
**Phylogenetic tree of the *Giardia duodenalis *assemblages constructed by neighbour-joining analysis, based on the nucleotide sequences of triosephosphate isomerase retrieved from this study compared with reference sequences of known assemblages from GenBank.** Bootstrap values obtained from 1000 replicates are indicated on branches in percentage.

Out of 110 microscopically-positive samples, 98 (89.1%) were successfully amplified based on analysis targeting *tpi* gene using nested-PCR assay. Sixty-two of the isolates were classified assemblage A, whereas 36 isolates were identified as assemblage B. The neighbour-joining tree placed four representative sequences [AF069556 (assemblage A), AF069557 (assemblage A), L02120 (assemblage A) and U57897 (assemblage A)] in one cluster with high bootstrap support. Phylogenetic analysis confirmed the monophyletic group of assemblage B (bootstrap = 100%) (Figure 1).

## Discussion

Molecular tools have been recently used to characterize the epidemiology of human giardiasis. Although isolates of *Giardia duodenalis* from human and various animals are morphologically similar, distinct host-adapted genotypes have been demonstrated within *G. duodenalis*[22,30]. Human giardiasis is cause by two distinct genetic groups of *G. duodenalis* known as assemblages A and B. Both assemblages are found associated with human infection globally and have also been detected in various animals. In this study, the results showed that all *G. duodenalis* infections in Orang Asli are due to assemblage A and assemblage B. This confirmed the results of a several local studies performed elsewhere [14,19].

At present, various molecular methods are available to distinguish these assemblages, mainly by nested-PCR followed by DNA sequencing or restriction fragment length polymorphism (RFLP), or by real-time PCR [31]. The majority of these assays are based on the amplification of a gene fragment with primers that bind to DNA sequences that are conserved in the two assemblages (or conserved in all *G. duodenalis* assemblages or in *Giardia* species). In the course of this study, triosephosphate isomerase (*tpi*) gene was specially chosen because of the high genetic heterogeneity displayed by *Giardia* species at this locus, as depicted by Thompson and Monis [7]. A recent local study done by Huey *et al.*[19] reported that the *tpi* gene achieved the highest percentage of amplicons produced (70%), followed by glutamate dehydrogenase (*gdh*) (45%) and beta-giardin (*bg*) (33%). Similar occurrences were also reported in previous studies [32,33]. Furthermore, according to the results of Sulaiman *et al.*[22], the *tpi* gene is a good phylogenetic marker for analysis of the molecular evolutionary and taxanomic relationship of *G. duodenalis*.

The distribution of *G. duodenalis* assemblages varied in different geographical areas. In the present study, sequences analysis of the 98 samples recovered from Orang Asli revealed 10.2% (62/611) *G. duodenalis* assemblage A and 5.9% (36/611) assemblage B, which were differs from previous local studies carried out by Mohammed Mahdy *et al.*[34] and Huey *et al.*[19]. In their studies, they found out that majority of these communities were infected with assemblage B. A high prevalence rate of assemblage A in the present study was in agreement with the results of Hussein *et al.*[35] which observed that assemblage A was more prevalent in eight stool samples. In Egypt, Helmy *et al.*[36] reported 75% assemblage A and 19.5% assemblage B among 41 patients. Another study conducted in Mexico had also shown that all 26 homogeneous isolates from humans belonged to assemblage A [37]. Similarly, all seven human isolates in Korea characterized at the SSU rRNA locus were from assemblage A [38]. Souza *et al.*[39] indicated that infections with assemblage A (78.4%) was more prevalent in Brazil, whereby Homan and Mank [40] observed that assemblage A was more prevalent in patients between 8 to 60 years of age in the Netherlands.

In contrast, a high prevalence rate of assemblage B was reported by Hatam-Nahavandi *et al.*[41]. Likewise, Tungtrongchitr *et al.*[42] in Thailand proved that assemblage B (51%) was more common than assemblage A (8%). Breathnach *et al.*[43] observed 73% of assemblage B and 24% assemblage A in 199 human stool samples in the southwest of London. In Nepal, Singh *et al.*[44] indicated that infection with assemblage B (74%) was more prevalent than assemblage A (20%) in patients older than 12 years of age. Curiously, Volotao *et al.*[45] did not find *G. duodenalis* assemblage B infecting humans in the city of Rio de Janeiro, Brazil. Results from each of these studies are not strictly comparable since amplifications were done on different *G. duodenalis* genes. Differences of *G. duodenalis* assemblage among the studied populations could be due to different modes of transmission in each area, comprising human-to-human, foodborne, waterborne or zoonotic transmissions.

Results of this study indicate a non-significant difference in the prevalence of *G. duodenalis* assemblages A and B infections between genders. Similar findings were observed by Gelanew *et al.*[46] and Anthony *et al.*[47] which found no significant difference between assemblage’s distribution and gender in Ethiopia and the Philippines. On the other hand, Mohammed Mahdy *et al.*[48] demonstrated that females were at two-fold higher risk of acquiring giardiasis caused by assemblage B compared to males.

Interestingly, this study showed that children age less than 15 years were at higher risk of being infected with assemblage B. This finding was in agreement with Mohammed Mahdy *et al.*[48] and Sadek *et al.*[49] that addressed this age group as a high risk group for giardiasis. This result was also consistent with worldwide reports including Malaysia suggesting that giardiasis is one of the major health problems among population of younger age groups [13,34,50]. The strong association between assemblage B infection and younger age group raises a question of whether *G. duodenalis* assemblages show age-specific pattern. Compared to previous studies carried out in Ethiopia [49], Philippines [50] and Australia [51], it seems that children are susceptible to both assemblages with variability in predominance from one country to country. The susceptibility could be attributed to the practicing improper personal hygiene. Lacking in effective immunity has also been postulated to explain this age-specific pattern.

Foodborne transmission of giardiasis was suggested in 1920s [52,53] and anecdotal evidence from outbreaks frequently implicated food handlers and contaminated vegetables or fruits [54]. Consuming raw vegetables was found to be a significant risk factor for *G. duodenalis* assemblage B in the present study. The most common vegetables consumed in Orang Asli were tapioca shoots, wild fern shoots and locally planted leaves. It was believed that this association was due to eating these vegetables with contaminated hands or hands that were insufficiently washed. Contaminated hands have been implicated to play a major role in the faecal-oral transmission of the communicable faecal-oral transmitted diseases in developing countries [55,56]. Other sources of food contamination include washing salad vegetables in water containing infectious cysts, the use of excrement (night soil) for fertilizer, contaminated irrigation water in the cultivation of food crops and the dissemination of cysts from stool to food by filth flies [57,58]. Vegetables can be surface contaminated with *Giardia* cysts [59,60], although it was often unclear whether cyst contamination was due to cultivation practices, contaminated water, insects or other animal vectors, or following handling by individuals with cyst-contaminated hands. Raw wastewater used as fertilizer but not treated wastewater or fresh water was responsible for contaminating coriander, mint, carrots and radish in a study conducted in Marrakech, Morocco [61].

The host distribution of *G. duodenalis* assemblage B was predominantly human and to a much lesser extent dog and wildlife. The present finding suggests that humans are the major source of assemblage B and it indicates the possibility of infected family members as the source of infection and direct transmission occurring within the household. It has also been demonstrated in Bangkok where human-to-human transmission of assemblage B was found among humans in temple communities [62]. Likewise, Cooper *et al.*[63] suggested that human-to-human transmission was much more important than animal-to-human transmission. In their study, they found that assemblage B frequently co-existed within families and co-infected subjects. Under such circumstances, children may be at constant risk of infection and this can be observed in the present study where the prevalence of *G. duodenalis* assemblage B was high in children younger than 15 years old. Furthermore, the prevalence of *G. duodenalis* assemblage B which indicates an anthroponotic transmission cycle has been seen in other countries in Asia [64] and the Indian subcontinent [65].

Zoonotic transmission of *G. duodenalis* is still under debate and despite increasing knowledge of the molecular identification of *Giardia* from different host species; the zoonotic potential of *G. duodenalis* is not clear [66,67]. However, the present epidemiological study once again highlighted close contact with household pets was 2.6 times more likely to become infected with *G. duodenalis* assemblage A. We considered household pets as dogs and cats that were kept contained in residence area (house and/or yard) for at least 12 h a day and allowed in the streets part of the day, either alone or accompanied by their owners. The role of dogs and cats as a definitive *G. duodenalis* host has been widely studied and recognized as being a public health problem, especially in developing countries and communities that were socioeconomically disadvantages as the one used in this study. In these communities, poor levels of hygiene and overcrowding, together with a lack of veterinary attention and zoonotic awareness, exacerbates the risk of giardiasis transmission [68]. Further evidence for zoonotic transmission was supported by the recovery of genetically similar isolates of *Giardia* in dogs and humans living within the same household, although it would appear that the risk of dog-human transmission was low.

In a recent report from Germany, it was found that of 60 *Giardia* positive samples collected from dogs in urban areas, 60% were infected with zoonotic *Giardia* from assemblage A, 12% with dog specific assemblages C and D and the remaining 28% harboured mixed infections [69]. Few studies have also been undertaken in cats but Vasilopulos *et al.*[70] examined 250 cats from Mississippi and Alabama and of 17 positive for *Giardia*, six infected with assemblage A and 11 with assemblage F (the cat genotype). Based on these findings, we believe that there are two transmission cycles in dogs; (i) the normal cycles between dogs which involves transmission of *G. duodenalis* cysts that belong to assemblages C or D and (ii) the other cycler includes cross transmission of *Giardia* from humans belonging to assemblage A [71] that grow in dog intestine. If this is true as the evidence supports, then dogs are *G. duodenalis* reservoir and transmit cysts in at least two ways which are from dog-to-dog and from humans-to-dogs and perhaps from dogs-to-humans.

It is interesting to note that mixed infections with *G. duodenalis* assemblages A and B were not detected in the current study. Similarly, Bertrand *et al.*[72] also did not found any mixed infections in their study among patients in France. However, the occurrence of mixed infections has been reported in molecular-based surveys performed in Australia [46], Italy [73], India [74] and United Kingdom [75]. The percentage of mixed infections ranged from 2 to 21% and was higher in less economically developed countries. Mixed infections can happen when a host ingests *Giardia* cysts of different genetic profiles or subsequent infection of an infected host by genetically different *Giardia* cysts. This was especially common in areas where giardiasis was endemic [8,76,77].

The present study however has several limitations. Firstly, direct amplification of cysts DNA from stool samples help to sole important questions such as presence of mixed infections, association between assemblages and host (pathogenicity) and selection for irrelevant genotypes during cultivation [22,78,79]. But using directly stool for DNA amplification cause to decrease the yield of DNA extracted that can be improved by apply a more effective approach. In addition, there are many PCR inhibitors (*i.e.* lipids, haemoglobin, bile salts and polysaccharides from mucus, bacteria and food degradation product) which can affect the result of amplification. For this reason, some extraction and amplification methods have been improved to develop more sensitive assays to identify gene. In some studies, specific DNA was detected at all target concentrations, demonstrating that QIAamp DNA kit extraction method could effectively remove PCR inhibitory substances [80,81]. Secondly, molecular techniques based on PCR in combination with techniques such as RFLP have been successfully used for differentiation of *G. duodenalis* up to sub-assemblages [79,82]. The *G. duodenalis* assemblage A isolates have been further grouped into sub-assemblages I and II, whereby the assemblage B isolates have been divided into sub-assemblages III and IV [83,84]. This, together with our results showed that it is important to combine different genotyping methods to get a clearer view of the assemblage of a *Giardia* isolate since different methods can group isolates into different assemblages and the resolution of sub-assemblages is dependent on the selected method.

## Conclusions

In conclusion, determination of the *G. duodenalis* assemblage is a useful way to understand the dynamic transmission of *Giardia* infection in Orang Asli. In the base of our findings, an anthroponotic origin of the infection route is suggested and underscored the fact that human are the main source of infection for assemblage B while close contact with domestic animals played a major role in the transmission for assemblage A. Because of the possibility of zoonotic transmission and the potential of household pets for hosting the parasite suggested by some researchers, further studies with a variety species of animal stool samples are recommended. Further studies using additional, more highly variable loci will provide more definitive evidence of both anthroponotic and zoonotic transmission in this community.

## Competing interests

The authors hereby declare that they have no competing interests.

## Authors’ contributions

TSA was involved in all phases of the study, including study design, data collection, data analysis and write up of the manuscript; NM supervised the study, and revised the manuscript; NM was involved in the statistical analysis of data; FMS and SNA were involved in the collection and laboratory examination of samples. All authors read and approved the final manuscript. TSA and NM are the guarantors of the paper.

## Pre-publication history

The pre-publication history for this paper can be accessed here:

http://www.biomedcentral.com/1471-2334/14/78/prepub
